# Clinical Outcomes Following Hemodynamic Parameter or Intravascular Imaging-Guided Percutaneous Coronary Intervention in the Era of Drug-Eluting Stents: An Updated Systematic Review and Bayesian Network Meta-Analysis of 28 Randomized Trials and 11,860 Patients

**DOI:** 10.3389/fcvm.2022.860189

**Published:** 2022-06-03

**Authors:** Meng-Jin Hu, Jiang-Shan Tan, Lu Yin, Yan-Yan Zhao, Xiao-Jin Gao, Jin-Gang Yang, Yue-Jin Yang

**Affiliations:** State Key Laboratory of Cardiovascular Disease, Fuwai Hospital, National Center for Cardiovascular Diseases, Chinese Academy of Medical Sciences and Peking Union Medical College, Beijing, China

**Keywords:** percutaneous coronary interventions (MeSH: D062645), drug-eluting stent (DES), coronary angiography, intravascular ultrasound (IVUS), optical coherence tomography (OCT), fractional flow reserve (FFR)

## Abstract

**Background:**

Coronary angiography (CAG) is the standard imaging modality for guiding percutaneous coronary interventions (PCI). Intracoronary imaging techniques such as intravascular ultrasound (IVUS) and optical coherence tomography (OCT), and hemodynamic parameter like fractional flow reserve (FFR) can overcome some limitations of CAG.

**Objective:**

We sought to explore the clinical outcomes of different PCI guidance modalities in the era of drug-eluting stent (DES).

**Methods:**

A network meta-analysis of 28 randomized trials and 11,860 patients undergoing different modalities-guided PCI in the era of DES was performed. Odds ratio (OR) with 95% credible interval (CrI) were calculated.

**Results:**

In comparison with CAG, IVUS was associated with a significant reduction in major adverse cardiovascular events (MACE, *OR*: 0.60; 95% *CrI*: 0.46–0.79), cardiovascular death (*OR*: 0.46; 95% *CrI*: 0.20–0.94), target vessel/lesion revascularization (TVR/TLR, *OR*: 0.55; 95% *CrI*: 0.41–0.74), and a trend toward decreased risk of stent thrombosis (*OR*: 0.44; 95% *CrI*: 0.17 to 1.00). FFR/quantitative flow ratio (QFR) could significantly reduce stroke compared with CAG, IVUS, and OCT/optical frequency domain imaging (OFDI). However, myocardial infarction (MI), all-cause death, stent thrombosis, and any revascularization presented similar risks for different PCI guidance modalities.

**Conclusion:**

In the era of DES, IVUS led to lower risks of MACE than CAG, which was mainly due to lower risks of cardiovascular death and TVR/TLR. A trend toward decreased risk of stent thrombosis was also observed with IVUS. Hemodynamic parameter (FFR/QFR)-guided PCI could significantly reduce the stroke risk compared with CAG, IVUS, and OCT/OFDI.

**Systematic Review Registration:**

[https://www.crd.york.ac.uk/PROSPERO/], identifier [CRD42021291442].

## Introduction

Coronary angiography (CAG) is the traditional and most widely used invasive imaging modality for guiding percutaneous coronary intervention (PCI). However, the two-dimensional projection of CAG cannot depict the structure of complex 3-dimensional arterial vessel wall, and thus evaluate the vessel dimensions and plaque characteristics, nor directly assess the result of stent implantation. Instead, intracoronary imaging through intravascular ultrasound (IVUS) and optical coherence tomography (OCT) can provide valuable incremental information that can be used clinically to optimize the stent implantation and minimize the stent-related problems (1–3). Fractional flow reserve (FFR) is a lesion-specific physiological index to evaluate the functional significance of coronary stenosis, and its benefit in guiding PCI has been proven by many clinical studies (4–6).

Although numerous meta-analyses and randomized trials have been published to compare the clinical outcomes between CAG and IVUS (1, 7, 8), CAG and OCT (9, 10), CAG and FFR (11, 12), yet just a few network meta-analyses are designed to compare the effects of all available modalities [CAG, IVUS, OCT/optical frequency domain imaging (OFDI), and FFR] for the guidance of PCI within a single analytical framework (13, 14). Moreover, randomized trials performed in the era of bare-metal stents (BMS) were also included in the aforementioned network meta-analyses, which may not be applicable to current clinical practice where drug-eluting stents (DES) have been widely used (15, 16). Pharmacological therapeutics have undergone great changes from BMS to DES era, especially the development of proprotein convertase subtilisin-kexin type 9 (PCSK-9) inhibitors, which can reduce levels of low-density lipoprotein cholesterol (LDL-C) by 50–70% when added to statins (17). Additionally, the previous network meta-analysis may be influenced by including observational studies. As more randomized trials and modalities have become available on PCI guidance, an updated comprehensive network meta-analysis of randomized trials is needed to evaluate the clinical outcomes associated with hemodynamic parameter (FFR or FFR related) or intravascular imaging (IVUS, OCT, or OFDI related)-guided PCI compared with CAG-guided PCI in the era of DES.

## Methods

This network meta-analysis was conducted according to the PRISMA (preferred reporting items for systematic reviews and meta-analyses) network meta-analysis extension statement (18). The summary data were obtained from the published randomized trials with approval from the respective institutional review committees. Therefore, no further sanction was required for our network meta-analysis. This meta-analysis has been registered at the PROSPERO international prospective register of systematic reviews (CRD42021291442).

## Search Strategy

We conducted a systematic search of the literature on October 12, 2021. The databases included Cochrane Central Register of Controlled Trials (CENTRAL), MEDLINE, EMBASE, and Web of Science. We also searched TCTMD, ClinicalTrials.gov, and major congress proceedings to identify potential studies. The medical subject headings or keywords included the following: coronary angiography, CAG; intravascular ultrasound, IVUS; optical coherence tomography, OCT; optical frequency domain imaging, OFDI; fractional flow reserve, FFR; instantaneous wave-free ratio, iFR; quantitative flow ratio, QFR; percutaneous coronary intervention, PCI; randomized controlled trial, RCT. The research syntax has been provided in Supplementary Table 1. Moreover, relevant randomized trials from reference lists of identified systematic reviews, meta-analyses, and relevant reviews were additionally hand searched to supplement the search of the electronic databases.

## Selection Criteria and Data Extraction

We included all randomized trials that compared any combination of the four category modalities: hemodynamic parameter-guided PCI [FFR, instantaneous wave-free ratio (iFR), quantitative flow ratio (QFR)], IVUS-guided PCI, OCT/OFDI-guided PCI, and CAG-guided PCI with DES implantation. Randomized trials without reporting our interested clinical outcomes were excluded. When multiple publications from the same randomized trial existed, we included the publication with the longest follow-up duration. The selection and data extraction processes were carried out in duplicate by two independent reviewers (HU MJ and GAO XJ), and any disagreement was resolved by consensus with a third-party reviewer (YANG JG).

## Quality Assessment of Risk of Bias

The risk of bias for all included studies was assessed using the Cochrane risk of bias assessment tool (19). Publication bias was investigated with comparison adjusted funnel plots.

## Outcomes

Our primary outcomes were major adverse cardiovascular events (MACE), cardiovascular death, myocardial infarction (MI), and target vessel/lesion revascularization (TVR/TLR) as reported by the trial authors. Secondary outcomes included all-cause death, stroke, stent thrombosis, and any revascularization. The definition of clinical outcomes was prescribed according to each randomized trial and can be found in Supplementary Table 2.

## Statistical Analysis

The Bayesian network meta-analysis was performed with a random effects model. Outcomes were reported as odds ratio (*OR*) with 95% credible interval (*CrI*) for all outcomes of interest. Four Markov chains were run simultaneously with 100,000 simulated draws after a burn-in of 10,000 iterations. We evaluated consistency with a node-splitting technique that compared the direct and indirect estimates for each comparison. The surface under the cumulative ranking curve (SUCRA) metric was used to compare the hierarchy of clinical outcomes of different PCI guidance modalities. SUCRA values vary between 0 and 100%, the higher the value, the higher the likelihood that a modality is in the top rank or highly effective (20). All analyses were conducted using R software (version 3.4.3) equipped with the “gemtc” package.

## Results

### Characteristics of Included Studies and Bias Assessment

**FIGURE 1:**
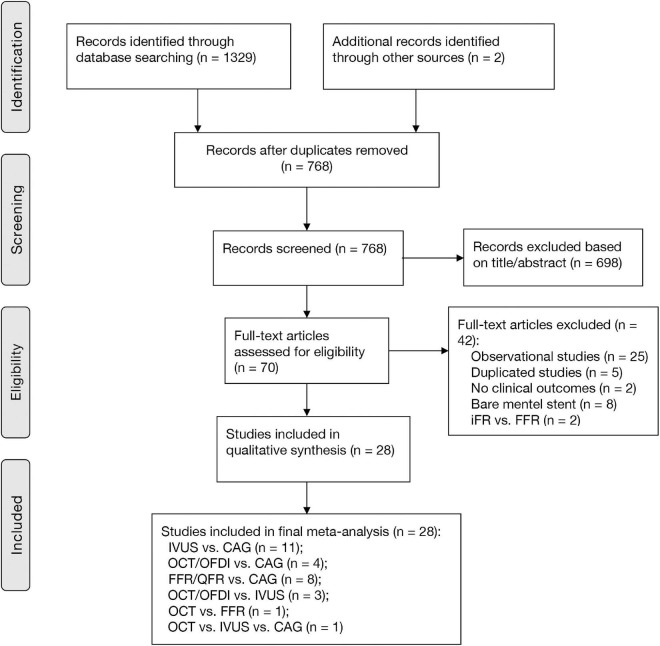
PRISMA diagram for study inclusion. CAG, coronary angiography; FFR, fractional flow reserve; IVUS, intravascular ultrasound; OCT, optical coherence tomography; OFDI, optical frequency domain imaging; QFR, quantitative flow ratio.

Figure 1 shows that a total of 1,329 citations met the search criteria, and the full text of 70 potentially eligible articles was scrutinized, resulting in including 28 randomized trials from 2010 to 2021 and 11,860 participants. Overall, eleven studies were comparisons between IVUS and CAG (1, 2, 8, 21–28), four studies were comparisons between OCT/OFDI and CAG (3, 10, 29, 30), eight studies were comparisons between FFR/QFR and CAG (4–6, 12, 31–34), three studies were comparisons between OCT/OFDI and IVUS (35–37), one study was comparison between OCT and FFR (38), one study was comparison among OCT, IVUS, and CAG (39), A total of 3,645 participants were randomly assigned to CAG, 3,396 to IVUS, 1,041 to OCT/OFDI, 3,778 to FFR/QFR (Table 1). The definition of clinical outcomes for each randomized trial can be found in Supplementary Table 1. The network evidence plots for all outcomes of interest are shown in Supplementary Figure 1. The risk of bias assessment was performed for each randomized trial and summarized in Supplementary Figure 2. Most of the studies were in the lowest categories for risk of bias: random sequence generation (25/28), allocation concealment (26/28), blinding of outcome assessment (20/28), incomplete outcome data (28/28), selective reporting (28/28), and other bias (terminated early, 25/28). However, blinding of participants and personnel (9/28) was in the highest category for risk of bias. The funnel plots of publication bias are shown in Supplementary Figure 3. Visual analysis of funnel plots demonstrated no evidence of publication bias.

**TABLE 1:** Baseline characteristics of included randomized trials.

Study/References	Location	Number of patients	Multicenter center	Primary endpoint	Mortality reported	Maximum follow-up	Lost to follow-up (%)
**IVUS versus CAG**							
Jakabcin et al. (21)	Czech Republic	105/105	NO	MACE	YES	1.5 years	NA
AVIO, (22)	International	142/142	YES	post-procedure in lesion minimal lumen diameter	NO	2 years	3.2
RESET, (23)	Korea	269/274	YES	MACE	YES	1 year	0
MOZART, (24)	Brazil and Spain	41/42	NA	total volume contrast agent used	YES	4 months	0
IVUS-XPL, (25)	Korea	700/700	YES	MACE	NO	1 year	5.0
CTO-IVUS, (8)	Korea	201/201	YES	cardiac death	YES	1 year	0.2
Tan et al. (26)	China	61/62	NO	MACE	NO	2 years	NA
AIR-CTO, (1)	China	115/115	YES	in-stent late lumen loss	YES	2 years	1.7
Wang et al. (27)	China	38/42	NO	MACE	NO	1 year	0
ULTIMATE, (2)	China	724/724	YES	target-vessel failure	YES	1 year	0.3
SURF, (28)	Australia	688/700	NO	Major bleeding and MACE	YES	30 days	3.4
**OCT/OFDI versus CAG**							
DOCTORS, (10)	France	120/120	YES	FFR at the end of the procedure	YES	6 months	0.4
ROBUST, (29)	Czech Republic	105/96	YES	MACE	YES	9 months	11.4
OPTICO BVS, (30)	Europe	19/19	YES	in-scaffold minimal lumen area	YES	6 months	0
OPTIMUM, (3)	Japan	56/54	YES	percentage of malapposed struts	YES	1 year	4.5
**FFR/QFR versus CAG**							
FAME, (31)	International	509/496	YES	MACE	YES	5 years	13.9
DKCRUSH-VI, (12)	China	160/160	YES	MACE	YES	1 year	0
FAMOUS–NSTEMI, (4)	United Kingdom	176/174	YES	medical management	YES	1 year	0
DEFER-DES, (5)	Korea	114/115	YES	MACE	NO	5 years	3.5
Zhang et al. (32)	China	110/110	NO	MACE	YES	1 year	NA
Quintella et al. (6)	Brazil	34/35	NO	MACE	YES	<12 months	1.4
FLOWER-MI, (33)	France	586/577	YES	MACE	YES	1 year	0.4
FAVOR III China, (34)	China	1913/1912	YES	MACE	YES	1 year	0.5
**OCT/OFDI versus IVUS**							
Habara et al. (35)	Japan	35/35	NO	stent expansion	YES	in-hospital	0
OPINION, (36)	Japan	412/405	YES	target vessel failure	NO	1 year	1.2
MISTIC-1, (37)	Japan	54/55	YES	in-segment minimum lumen area	YES	3 years	0.9
**OCT versus FFR**							
FORZA, (38)	Italy	174/176	NO	MACE	YES	13 months	NA
**OCT versus IVUS versus CAG**							
ILUMIEN III, (39)	International	158/146/146	YES	minimal stent area	YES	1 year	4.2

*ACS, acute coronary syndrome; CAG, coronary angiography; FFR, fractional flow reserve; IVUS, intravascular ultrasound; MACE, major adverse cardiac events; OCT, optical coherence tomography; OFDI, optical frequency domain imaging; QFR, quantitative flow ratio.*

### Primary Outcomes

#### Major Adverse Cardiovascular Events

**FIGURE 2:**
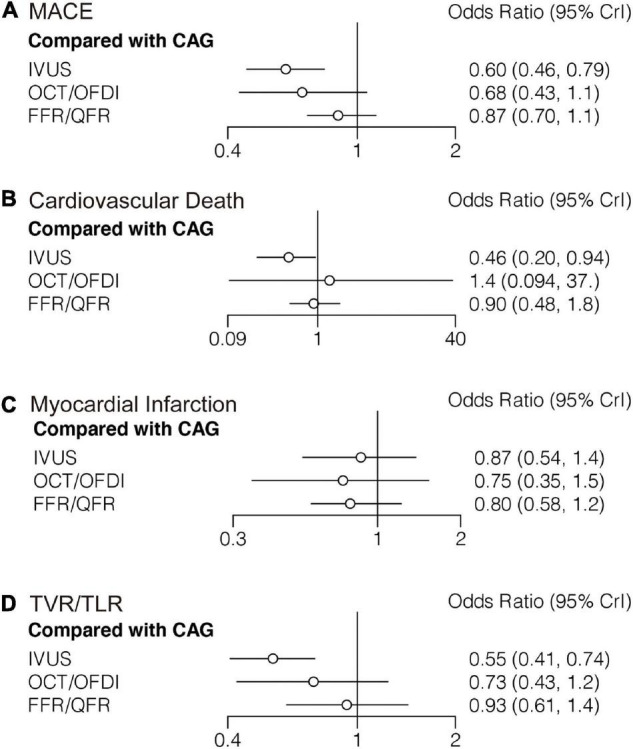
Comparisons of primary outcomes among guidance modalities included in the network meta-analysis. CAG, coronary angiography; FFR, fractional flow reserve; IVUS, intravascular ultrasound; OCT, optical coherence tomography; OFDI, optical frequency domain imaging; QFR, quantitative flow ratio. **(A)** Major adverse cardiovascular events. **(B)** Cardiovascular death. **(C)** Myocardial infarction. **(D)** target vessel revascularization/target lesion revascularization.

**FIGURE 3:**
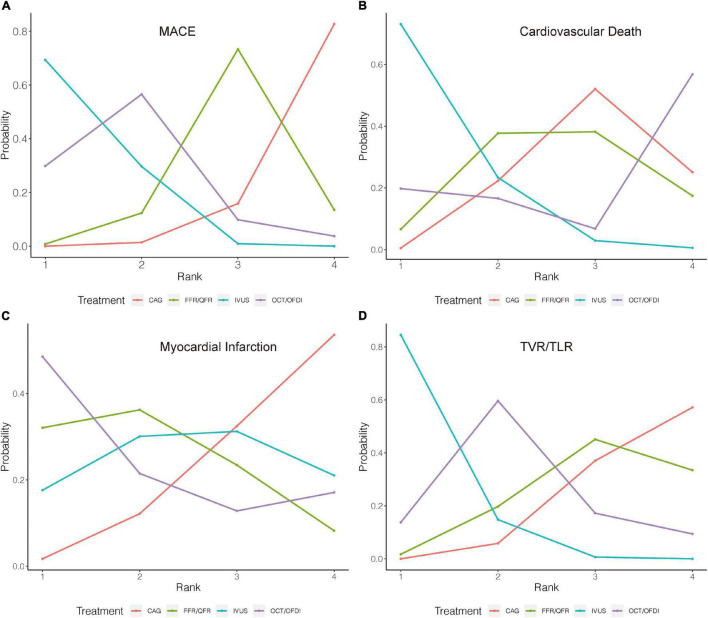
Rank probability analysis for primary outcomes among guidance modalities included in the network meta-analysis. Rank 1 is the best while rank 4 is the worst. For example, for MACE **(A)**, IVUS had the highest probability of ranking 1, CAG had the lowest probability of ranking 1. CAG, coronary angiography; FFR, fractional flow reserve; IVUS, intravascular ultrasound; OCT, optical coherence tomography; OFDI, optical frequency domain imaging; QFR, quantitative flow ratio. **(A)** Major adverse cardiovascular events. **(B)** Cardiovascular death. **(C)** Myocardial infarction. **(D)** Target vessel revascularization/target lesion revascularization.

Twenty-two randomized trials (14,876 patients) reported 1,199 (8.06%) MACE events as classified by individual trial definitions. In comparison with CAG, IVUS was associated with a significant reduction in MACE (*OR*: 0.60; 95% *CrI*: 0.46–0.79), whereas MACE was not significantly reduced with OCT/OFDI or FFR/QFR (Figure 2A). There were no significant differences observed in the left guidance modality comparisons (Table 2). Figure 3A demonstrated that IVUS had high rankings (low likelihoods) for causing MACE.

**TABLE 2:** Comparisons of primary outcomes among guidance modalities included in the network meta-analysis.

	OR (95% CrI)	OR (95% CrI)	OR (95% CrI)
**MACE**			
CAG	0.60 (0.46, 0.79)	0.68 (0.43, 1.10)	0.87 (0.70, 1.10)
	IVUS	1.11 (0.72, 1.73)	1.44 (1.03, 2.08)
		OCT/OFDI	1.30 (0.82, 2.07)
			FFR/QFR
**Cardiovascular death**			
CAG	0.46 (0.20, 0.94)	1.40 (0.09, 37.00)	0.90 (0.48, 1.80)
	IVUS	2.96 (0.20, 77.72)	1.96 (0.73, 5.96)
		OCT/OFDI	0.66 (0.02, 11.01)
			FFR/QFR
**Myocardial infarction**			
CAG	0.87 (0.54, 1.40)	0.75 (0.35, 1.50)	0.80 (0.58, 1.20)
	IVUS	0.86 (0.41, 1.79)	0.92 (0.54, 1.76)
		OCT/OFDI	1.07 (0.52, 2.38)
			FFR/QFR
**TVR/TLR**			
CAG	0.55 (0.41, 0.74)	0.73 (0.43, 1.20)	0.93 (0.61, 1.40)
	IVUS	1.33 (0.79, 2.22)	1.68 (1.02, 2.83)
		OCT/OFDI	1.26 (0.68, 2.44)
			FFR/QFR

#### Cardiovascular Death

Sixteen trials (10,985 patients) reported 123 (1.12%) cardiovascular death events. In comparison with CAG, IVUS could significantly reduce cardiovascular death (*OR*: 0.46; 95% *CrI*: 0.20–0.94), whereas OCT/OFDI or FFR/QFR showed no significant difference (Figure 2B). There were also no significant differences among the left guidance modality comparisons (Table 2). Similarly, IVUS had high rankings (low likelihoods) for causing cardiovascular death (Figure 3B).

#### Myocardial Infarction

Twenty-six randomized trials (15,643 patients) reported 527 (3.37%) MI events. In comparison with CAG, there were no significant differences with IVUS, OCT/OFDI, or FFR/QFR (Figure 2C). In addition, no significant differences among the left guidance modality comparisons were observed (Table 2). However, OCT/OFDI had high rankings (low likelihoods) for causing MI, whereas CAG had low rankings (high likelihoods) for causing MI (Figure 3C).

#### Target Vessel/Lesion Revascularization

Twenty-two trials (12,810 patients) reported 379 (2.96%) TVR/TLR events. In comparison with CAG, IVUS was associated with a significant reduction of TVR/TLR (*OR*: 0.55; 95% *CrI*: 0.41–0.74), whereas OCT/OFDI or FFR/QFR were not (Figure 2D). There were also no significant differences among the left guidance modality comparisons (Table 2). IVUS had high rankings (low likelihoods) for causing TVR/TLR (Figure 3D).

### Secondary Outcomes

#### All-Cause Death

**FIGURE 4:**
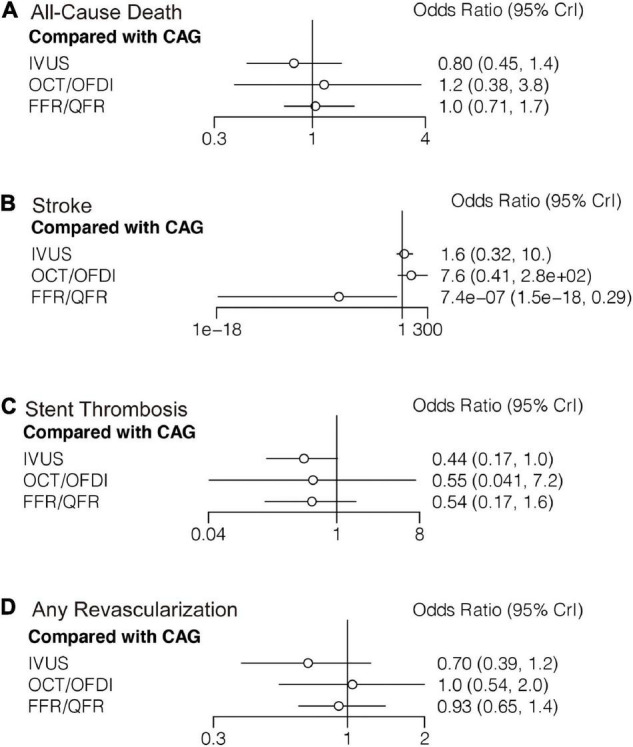
Comparisons of secondary outcomes among guidance modalities included in the network meta-analysis. CAG, coronary angiography; FFR, fractional flow reserve; IVUS, intravascular ultrasound; OCT, optical coherence tomography; OFDI, optical frequency domain imaging; QFR, quantitative flow ratio. **(A)** All-cause death. **(B)** Stroke. **(C)** Stent thrombosis. **(D)** Any revascularization.

**FIGURE 5:**
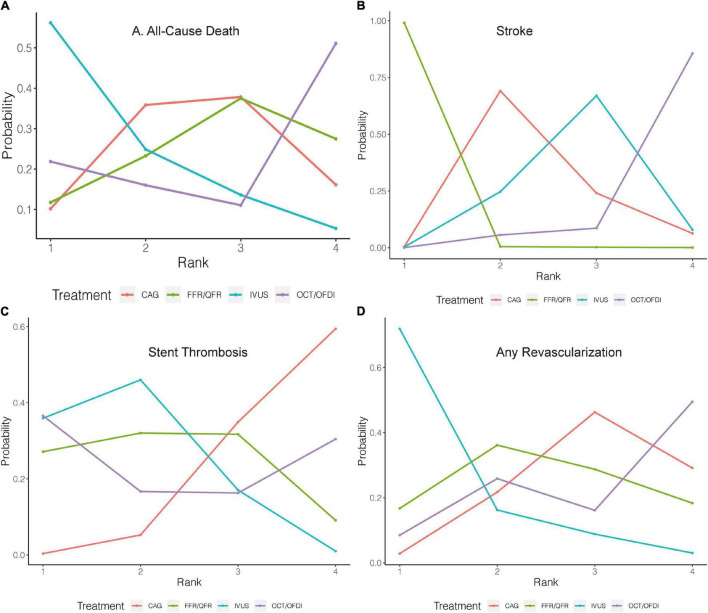
Rank probability analysis for secondary outcomes among guidance modalities included in the network meta-analysis. Rank 1 is the best while rank 4 is the worst. For example, for all-cause death **(A)**, IVUS had the highest probability of ranking 1, CAG had the lowest probability of ranking 1. CAG, coronary angiography; FFR, fractional flow reserve; IVUS, intravascular ultrasound; OCT, optical coherence tomography; OFDI, optical frequency domain imaging; QFR, quantitative flow ratio. **(A)** All-cause death. **(B)** Stroke. **(C)** Stent thrombosis. **(D)** Any revascularization.

Twenty-two randomized trials (12,768 patients) reported 246 (1.93%) cases of all-cause death. In comparison with CAG, there were no significant differences with IVUS, OCT/OFDI, or FFR/QFR in terms of all-cause death (Figure 4A). Similarly, no significant differences were observed among the left guidance modality comparisons (Table 3). However, IVUS had high rankings (low likelihoods) for causing all-cause death (Figure 5A).

**TABLE 3:** Comparisons of secondary outcomes among guidance modalities included in the network meta-analysis.

	OR (95% CrI)	OR (95% CrI)	OR (95% CrI)
**All-Cause death**			
CAG	0.80 (0.45, 1.40)	1.20 (0.38, 3.80)	1.00 (0.71, 1.70)
	IVUS	1.45 (0.48, 4.86)	1.32 (0.67, 2.66)
		OCT/OFDI	0.91 (0.28, 2.85)
			FFR/QFR
**Stroke**			
CAG	1.60 (0.32, 10.00)	7.60 (0.41, 280.93)	0 (0, 0.29)
	IVUS	4.53 (0.40, 110.00)	0 (0, 0.21)
		OCT/OFDI	0 (0, 0.06)
			FFR/QFR
**Stent thrombosis**			
CAG	0.44 (0.17, 1.00)	0.55 (0.04, 7.20)	0.54 (0.17, 1.60)
	IVUS	1.27 (0.10, 15.29)	1.23 (0.29, 5.24)
		OCT/OFDI	0.97 (0.05, 16.70)
			FFR/QFR
**Any revascularization**			
CAG	0.70 (0.39, 1.20)	1.00 (0.54, 2.00)	0.93 (0.65, 1.40)
	IVUS	1.48 (0.77, 2.96)	1.32 (0.68, 2.75)
		OCT/OFDI	0.89 (0.42, 1.91)
			FFR/QFR

#### Stroke

Six trials (4,214 patients) in total reported 17 (0.40%) stroke events. In comparison with CAG, FFR/QFR could significantly reduce stroke events (*OR*: 7.4e-07; 95% *CrI*: 1.5e-18–0.29), whereas IVUS or OCT/OFDI were not (Figure 4B). Moreover, FFR could significantly reduce stroke compared with both IVUS (*OR*: 0; 95% *CrI*: 0–0.21) and OCT/OFDI (*OR*: 0; 95% *CrI*: 0–0.06) (Table 3). Figure 5B revealed that FFR had high rankings (low likelihoods) for causing stroke.

#### Stent Thrombosis

Fifteen randomized trials (11,269 patients) reported 62 (0.55%) stent thrombosis. In comparison with CAG, IVUS had a trend to decrease stent thrombosis (*OR*: 0.44; 95% *CrI*: 0.17–1.00), while OCT/OFDI or FFR/QFR were not (Figure 4C). In addition, no significant differences were observed among the left guidance modality comparisons (Table 3). CAG had low rankings (high likelihoods) for causing stent thrombosis (Figure 5C).

#### Any Revascularization

Fifteen randomized trials (9,683 patients) reported 510 (5.27%) any revascularization events. In comparison with CAG, there were no significant differences with IVUS, OCT/OFDI, or FFR/QFR in terms of any revascularization (Figure 4D). There were also no statistically significant differences in any revascularization risk among the left guidance modality comparisons (Table 3). However, IVUS had high rankings (low likelihoods) for causing any revascularization (Figure 5D).

**FIGURE 6:**
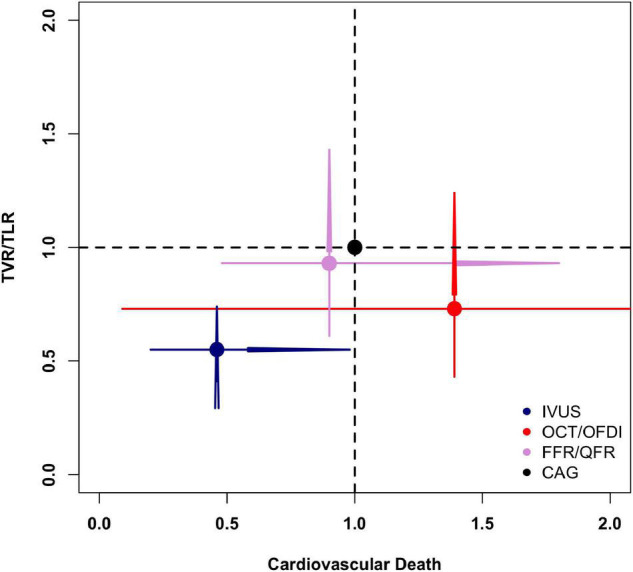
Comparisons of cardiovascular death and target vessel/lesion revascularization in the network meta-analysis. CAG, coronary angiography; FFR, fractional flow reserve; IVUS, intravascular ultrasound; OCT, optical coherence tomography; OFDI, optical frequency domain imaging; QFR, quantitative flow ratio; TVR/TLR, target vessel/lesion revascularization.

Figure 6 illustrates the risk of cardiovascular death versus TVR/TLR of different PCI guidance modalities compared with CAG. Overall, the results favored IVUS-guided PCI for fewer cardiovascular death and TVR/TLR compared with CAG.

### Network Coherence

The network node-split outcomes for MACE (Supplementary Figure 4A), cardiovascular death (Supplementary Figure 4B), MI (Supplementary Figure 4C), TVR/TLR (Supplementary Figure 4D), all-cause death (Supplementary Figure 4E), stroke (Supplementary Figure 4F), stent thrombosis (Supplementary Figure 4G), and any revascularization (Supplementary Figure 4H) revealed that there were no noticeable differences between direct and indirect estimates in closed loops that allowed the assessment of network coherence.

### Sensitivity Analysis

As a sensitivity analysis, results based on fixed effect model were also calculated and similar results were observed (Supplementary Figure 5).

## Discussion

In our network meta-analysis, which included 28 randomized trials and 11,860 patients, we analyzed the clinical outcomes of four category PCI guidance modalities (CAG, IVUS, OCT/OFDI, and FFR/QFR) in the era of DES, and the findings can be summarized as follows. Firstly, IVUS led to lower risks of MACE than CAG, which was mainly due to lower risks of cardiovascular death and TVR/TLR. A trend toward decreased risk of stent thrombosis was also observed with IVUS. Secondly, hemodynamic parameter (FFR/QFR)-guided PCI could significantly reduce stroke compared with CAG, IVUS, and OCT/OFDI. Thirdly, MI, all-cause death, stent thrombosis, and any revascularization presented similar risks for the four category PCI guidance modalities.

Similar with our findings, a meta-analysis including seven trials with 3192 patients in the era of DES also revealed that IVUS-guided PCI was associated with a reduction in the risk of MACE (6.5 versus 10.3%; *OR*: 0.60; 95% *CI*: 0.46–0.77), which was mainly because of reduction in the risk of TLR (4.1 versus 6.6%; *OR*: 0.60; 95% *CI*: 0.43–0.84). The risk of stent thrombosis (0.6 versus 1.3%; *OR*: 0.49; 95% *CI*: 0.24–0.99) also appeared to be lower in the IVUS-guided group, and there was a trend toward lower risk of cardiovascular mortality (0.5 versus 1.2%; *OR*: 0.46; 95% *CI*: 0.21–1.00). After all, IVUS allows for easier visualization of the entire vessel structure, particularly when extensive circumferential calcification or attenuated plaques are not encountered (14). Moreover, in the Assessment of Dual Antiplatelet Therapy with Drug-Eluting Stents (ADAPT-DES) study which enrolled an all-comers population, IVUS-guided PCI was also associated with lower rates of stent thrombosis, MI, and TVR/TLR compared with CAG-guided PCI. Compared with CAG-guided PCI, a larger stent or balloon and/or higher inflation pressures (that would minimize under-expansion) were used in approximately 60% of IVUS-guided procedures, and additional stents (that would mitigate inflow/out-flow issues) were used in about 20% of patients. These strategies are most likely responsible for the lower rates of stent thrombosis and TVR/TLR observed in the IVUS-guided cohort in the present study (40). The aforementioned studies also confirmed that IVUS-guided PCI was superior to CAG-guided PCI not only in selected patients from randomized trials but also in all-comers from real-world scenarios.

An expert consensus document of the European Association of Percutaneous Cardiovascular Interventions claimed that IVUS and OCT are equivalent (and superior to CAG) in guiding and optimizing most PCI procedures (41). In our meta-analysis, it is indeed that no significant differences were observed between IVUS and OCT/OFDI. However, IVUS could decrease the risks of MACE, cardiovascular death, and TVR/TLR compared with CAG, where OCT/OFDI could not. Due to lower tissue penetration, especially in lipid-rich tissue, OCT is limited in assessing plaque burden and detecting vessel size in the presence of diffuse disease, whereas IVUS is an approach used to guide stenting sizing. Moreover, OCT is frequently unable to visualize the ostium as proper blood clearance is probably a challenge. Also, blood clearance needed for image acquisition in OCT increases the radio-contrast burden, which is particularly detrimental in patients with renal disease, whereas IVUS can minimize the use of iodine contrast in PCI procedure (24, 42). All of the aforementioned characteristics may contribute to the positive prognosis associated with IVUS and negative prognosis associated with OCT/OFDI. Meanwhile, it is noteworthy that compared with IVUS with numerous randomized trials comparing IVUS-guided versus CAG-guided PCI, there is limited research evidence on OCT-guided versus CAG-guided PCI with respect to clinical outcomes and no RCT is powered for clinical outcomes. Therefore, the lack of significant difference between OCT-guided versus CAG-guided PCI may be a result of limited number of patients and underpowered for the outcomes of interest. In addition, IVUS has been used clinically for almost three decades and extensive clinical experience has been gained, which may translate into positive prognosis.

In our meta-analysis, decreased risk of stroke associated with hemodynamic parameter (FFR/QFR)-guided PCI was also observed. As revealed in the British Heart Foundation FAMOUS–NSTEMI randomized trial, the proportion of patients treated initially by medical therapy was higher in the FFR-guided group than in the CAG-guided group [40 (22.7%) versus 23 (13.2%), difference 95% *CI*: 1.4–17.7%, *p* = 0.022], whereas the aggressive coronary revascularization was higher in the CAG-guided PCI (86.8 versus 77.3%). In a propensity score matching study including a total of 1,299 patients with left ventricular ejection fraction ≤ 50% (433 FFR-guided PCI, 866 CAG-guided PCI), FFR-guided PCI was associated with a lower risk of stroke compared with CAG-guided PCI (0 versus 2%; *HR*: 0.84; 95% *CI*: 0.62–0.96) during 1-year follow-up, whereas the differences disappeared after 5-years follow-up (3 versus 3%; *HR*: 0.68; 95% *CI*: 0.37– 1.65) (43). Therefore, it seems that CAG-associated stroke was mainly confined during peri-procedural and short-term follow-up, possibly due to more aggressive treatments in the CAG-guided group. However, we have to admit the fact that in our meta-analysis, just six trials (4,214 patients) in total reported 17 (0.40%) stroke events, which was too small in scale and maybe the reason for wide *CrI*. Considering the limited number of randomized trials focusing on the issue of stroke, further randomized trials are warranted to validate the rationality of different modalities in guiding PCI in the era of DES. The currently enrolling ILUMIEN III trial (NCT03507777) will randomize between 2,490 and 3,656 patients with high-risk clinical characteristics (diabetes) and/or complex angiographic lesions to compare the clinical outcomes between OCT-guided versus CAG-guided PCI. The principal results are expected to be published in 2022, which will provide significant evidence on the role of OCT in the guidance of PCI (44).

Despite the better prognosis associated with IVUS, yet CAG is still the mostly used modality in clinical practice. Moreover, the drawbacks associated with DES should be acknowledged. For example, although the healing response was similar and neoatherosclerosis was low in patients receiving durable- or biodegradable-polymer (45), histopathology, and intravascular imaging have detected neoatherosclerosis earlier and more frequently with DES compared with BMS (45). However, with the advancement in medical management (46) and techniques (47), it is promising that the prognosis associated with cardiovascular disease will improve greatly.

## Limitations

The present study should be interpreted with caution in light of some limitations. First, this is a study-level meta-analysis providing average treatment effects. The absence of patient-level data prevents us from assessing the effect of baseline clinical characteristics in PCI guidance modalities which might affect clinical outcomes. Second, subgroup analysis based on stable or acute coronary symptom is impossible because both stable and acute coronary symptom patients were included in the same trial. However, the ADAPT-DES study revealed that IVUS-guided PCI was superior to CAG-guided PCI in both stable and acute coronary symptom patients (40). Third, just six trials (4,214 patients) in total reported 17 (0.40%) stroke events, which was too small in scale and maybe the reason for wide *CrI*. Therefore, more randomized trials are warranted to validate the rationality of different modalities in guiding PCI in the era of DES. Fourth, IVUS has been used clinically for almost three decades and extensive clinical experience has been gained. However, the same scenario was not obtained for other PCI guidance modalities (OCT, OFDI, FFR, and QFR). Considering the fact that a long learning curve is required to commend a new PCI guidance modality, therefore, unfamiliar with the newly developed PCI guidance modality may negatively affect prognosis.

## Conclusion

Our comprehensive network meta-analysis provides evidence that IVUS-guided PCI resulted in less MACE, cardiovascular death, and TVR/TLR. FFR/QFR-guided PCI resulted in decreased risk of stroke in the DES era. Further studies are still required to validate the rationality of different modalities in guiding PCI in the era of DES.

## Data Availability Statement

The original contributions presented in the study are included in the article/Supplementary Material, further inquiries can be directed to the corresponding author.

## Author Contributions

M-JH: conceptualization, investigation, methodology, resources, validation, visualization, writing – original draft, and writing – review and editing. J-ST: methodology, resources, validation, visualization, and writing – original draft. LY, Y-YZ, J-GY, and X-JG: data curation, formal analysis, methodology, software, and writing – original draft. Y-JY: formal analysis, funding acquisition, investigation, methodology, project administration, resources, supervision, validation, visualization, and writing – review and editing. All authors contributed to the article and approved the submitted version.

## Conflict of Interest

The authors declare that the research was conducted in the absence of any commercial or financial relationships that could be construed as a potential conflict of interest.

## Publisher’s Note

All claims expressed in this article are solely those of the authors and do not necessarily represent those of their affiliated organizations, or those of the publisher, the editors and the reviewers. Any product that may be evaluated in this article, or claim that may be made by its manufacturer, is not guaranteed or endorsed by the publisher.

## Supplementary Material

Supplementary Figure 1 Network evidence plot for major adverse cardiovascular events **(A)**, Cardiovascular death **(B)**, Myocardial infarction **(C)**, Target vessel/lesion revascularization **(D)**, All-cause death **(E)**, Stroke **(F)**, Stent thrombosis **(G)**, and Any revascularization **(H)**. CAG, coronary angiography; FFR, fractional flow reserve; IVUS, intravascular ultrasound; OCT, optical coherence tomography; OFDI, optical frequency domain imaging; QFR, quantitative flow ratio.

Supplementary Figure 2 Methodological quality assessment for each included randomized trial. +, low risk of bias; -, high risk of bias; ?, unclear risk of bias.

Supplementary Figure 3 Funnel plot of publication bias for major adverse cardiovascular events **(A)**, Cardiovascular death **(B)**, Myocardial infarction **(C)**, Target vessel/lesion revascularization **(D)**, All-cause death **(E)**, Stroke **(F)**, Stent thrombosis **(G)**, and Any revascularization **(H)**. CAG, coronary angiography; FFR, fractional flow reserve; IVUS, intravascular ultrasound; OCT, optical coherence tomography; OFDI, optical frequency domain imaging; QFR, quantitative flow ratio.

Supplementary Figure 4 Network node-split for major adverse cardiovascular events **(A)**, Cardiovascular death **(B)**, Myocardial infarction **(C)**, Target vessel/lesion revascularization **(D)**, All-cause death **(E)**, Stroke **(F)**, Stent thrombosis **(G)**, and Any revascularization **(H)**. CAG, coronary angiography; FFR, fractional flow reserve; IVUS, intravascular ultrasound; OCT, optical coherence tomography; OFDI, optical frequency domain imaging; QFR, quantitative flow ratio.

Supplementary Figure 5 Forest plot for major adverse cardiovascular events **(A)**, Cardiovascular death **(B)**, Myocardial infarction **(C)**, Target vessel/lesion revascularization **(D)**, All-cause death **(E)**, Stroke **(F)**, Stent thrombosis **(G)**, and Any revascularization **(H)** with a fixed effects mode. CAG, coronary angiography; FFR, fractional flow reserve; IVUS, intravascular ultrasound; OCT, optical coherence tomography; OFDI, optical frequency domain imaging; QFR, quantitative flow ratio.

## References

[1] Tian NL Gami SK Ye F Zhang JJ Liu ZZ Lin S Angiographic and clinical comparisons of intravascular ultrasound- versus angiography-guided drug-eluting stent implantation for patients with chronic total occlusion lesions: two-year results from a randomised AIR-CTO study. *EuroIntervention.* (2015) 10:1409–17. 10.4244/EIJV10I12A245 25912391

[2] Zhang J Gao X Kan J Ge Z Han L Lu S Intravascular ultrasound versus angiography-guided drug-eluting stent implantation: the ULTIMATE trial. *J Am Coll Cardiol.* (2018) 72:3126–37. 30261237 10.1016/j.jacc.2018.09.013

[3] Onuma Y Kogame N Sotomi Y Miyazaki Y Asano T Takahashi K A randomized trial evaluating online 3-dimensional optical frequency domain imaging-guided percutaneous coronary intervention in bifurcation lesions. *Circ Cardiovasc Interv.* (2020) 13:e009183. 10.1161/CIRCINTERVENTIONS.120.009183 33272034 PMC7732152

[4] Layland J Oldroyd KG Curzen N Sood A Balachandran K Das R Fractional flow reserve vs. angiography in guiding management to optimize outcomes in non-ST-segment elevation myocardial infarction: the British heart foundation FAMOUS-NSTEMI randomized trial. *Eur Heart J.* (2015) 36:100–11. 10.1093/eurheartj/ehu338 25179764 PMC4291317

[5] Park SH Jeon KH Lee JM Nam CW Doh JH Lee BK Long-term clinical outcomes of fractional flow reserve-guided versus routine drug-eluting stent implantation in patients with intermediate coronary stenosis: five-year clinical outcomes of DEFER-DES trial. *Circ Cardiovasc Interv.* (2015) 8:e002442. 10.1161/CIRCINTERVENTIONS.115.002442 26643736

[6] Quintella EF Ferreira E Azevedo VMP Araujo DV Sant’Anna FM Amorim B Clinical outcomes and cost-effectiveness analysis of FFR compared with angiography in multivessel disease patient. *Arq Bras Cardiol.* (2019) 112:40–7. 10.5935/abc.20180262 30570071 PMC6317625

[7] Bavishi C Sardar P Chatterjee S Khan AR Shah A Ather S Intravascular ultrasound-guided vs angiography-guided drug-eluting stent implantation in complex coronary lesions: meta-analysis of randomized trials. *Am Heart J.* (2017) 185:26–34. 10.1016/j.ahj.2016.10.008 28267472

[8] Kim BK Shin DH Hong MK Park HS Rha SW Mintz GS Clinical impact of intravascular ultrasound-guided chronic total occlusion intervention with zotarolimus-eluting versus biolimus-eluting stent implantation: randomized study. *Circ Cardiovasc Interv.* (2015) 8:e002592. 10.1161/CIRCINTERVENTIONS.115.002592 26156151

[9] Kuku KO Ekanem E Azizi V Melaku G Bui A Meirovich YF Optical coherence tomography-guided percutaneous coronary intervention compared with other imaging guidance: a meta-analysis. *Int J Cardiovasc Imaging.* (2018) 34:503–13. 10.1007/s10554-017-1272-2 29151138

[10] Meneveau N Souteyrand G Motreff P Caussin C Amabile N Ohlmann P Optical coherence tomography to optimize results of percutaneous coronary intervention in patients with non-ST-elevation acute coronary syndrome: results of the multicenter, randomized DOCTORS study (does optical coherence tomography optimize results of stenting). *Circulation.* (2016) 134:906–17. 10.1161/circulationaha.116.024393 27573032

[11] Bundhun PK Yanamala CM Huang F. Comparing the adverse clinical outcomes associated with fraction flow reserve-guided versus angiography-guided percutaneous coronary intervention: a systematic review and meta-analysis of randomized controlled trials. *BMC Cardiovasc Disord.* (2016) 16:249. 10.1186/s12872-016-0427-8 27912739 PMC5135818

[12] Chen SL Ye F Zhang JJ Xu T Tian NL Liu ZZ Randomized comparison of FFR-guided and angiography-guided provisional stenting of true coronary bifurcation lesions: the DKCRUSH-VI trial (double kissing crush versus provisional stenting technique for treatment of coronary bifurcation lesions VI). *JACC Cardiovasc Interv.* (2015) 8:536–46. 10.1016/j.jcin.2014.12.221 25819187

[13] Iannaccone M Abdirashid M Annone U Saint-Hilary G Meier P Chieffo A Comparison between functional and intravascular imaging approaches guiding percutaneous coronary intervention: a network meta-analysis of randomized and propensity matching studies. *Catheter Cardiovasc Interv.* (2020) 95:1259–66. 10.1002/ccd.28410 31400061

[14] Buccheri S Franchina G Romano S Puglisi S Venuti G D’Arrigo P Clinical outcomes following intravascular imaging-guided versus coronary angiography-guided percutaneous coronary intervention with stent implantation: a systematic review and Bayesian network meta-analysis of 31 studies and 17,882 patients. *JACC Cardiovasc Interv.* (2017) 10:2488–98. 10.1016/j.jcin.2017.08.051 29153502

[15] Yun KH Lee SY Cho BR Jang WJ Song YB Oh JH Safety of 3-month dual antiplatelet therapy after implantation of ultrathin sirolimus-eluting stents with biodegradable polymer (Orsiro): results from the SMART-CHOICE trial. *J Am Heart Assoc.* (2021) 10:e018366. 10.1161/JAHA.120.018366 PMC7955499 33345567

[16] Watanabe H Domei T Morimoto T Natsuaki M Shiomi H Toyota T Details on the effect of very short dual antiplatelet therapy after drug-eluting stent implantation in patients with high bleeding risk: insight from the STOPDAPT-2 trial. *Cardiovasc Interv Ther.* (2021) 36:91–103. 10.1007/s12928-020-00651-9 32086787

[17] Rodriguez-Gutierrez R Shah ND Montori VM. Predicting the overuse of PCSK-9 inhibitors. *JAMA.* (2015) 314:1909–10. 10.1001/jama.2015.13414 26547456

[18] Hutton B Salanti G Caldwell DM Chaimani A Schmid CH Cameron C The PRISMA extension statement for reporting of systematic reviews incorporating network meta-analyses of health care interventions: checklist and explanations. *Ann Intern Med.* (2015) 162:777–84. 10.7326/M14-2385 26030634

[19] Higgins JP Altman DG Gøtzsche PC Jüni P Moher D Oxman AD The Cochrane collaboration’s tool for assessing risk of bias in randomised trials. *BMJ.* (2011) 343:d5928. 10.1136/bmj.d5928 22008217 PMC3196245

[20] Salanti G Ades AE Ioannidis JP. Graphical methods and numerical summaries for presenting results from multiple-treatment meta-analysis: an overview and tutorial. *J Clin Epidemiol.* (2011) 64:163–71. 10.1016/j.jclinepi.2010.03.016 20688472

[21] Jakabcin J Spacek R Bystron M Kvasnák M Jager J Veselka J Long-term health outcome and mortality evaluation after invasive coronary treatment using drug eluting stents with or without the IVUS guidance. Randomized control trial. HOME DES IVUS. *Catheter Cardiovasc Interv.* (2010) 75:578–83. 10.1002/ccd.22244 19902491

[22] Chieffo A Latib A Caussin C Presbitero P Galli S Menozzi A A prospective, randomized trial of intravascular-ultrasound guided compared to angiography guided stent implantation in complex coronary lesions: the AVIO trial. *Am Heart J.* (2013) 165:65–72. 10.1016/j.ahj.2012.09.017 23237135

[23] Kim JS Kang TS Mintz GS Park BE Shin DH Kim BK Randomized comparison of clinical outcomes between intravascular ultrasound and angiography-guided drug-eluting stent implantation for long coronary artery stenoses. *JACC Cardiovasc Interv.* (2013) 6:369–76. 10.1016/j.jcin.2012.11.009 23523455

[24] Mariani J Jr Guedes C Soares P Zalc S Campos CM Lopes AC Intravascular ultrasound guidance to minimize the use of iodine contrast in percutaneous coronary intervention: the MOZART (Minimizing cOntrast utiliZation With IVUS Guidance in coRonary angioplasTy) randomized controlled trial. *JACC Cardiovasc Interv.* (2014) 7:1287–93. 10.1016/j.jcin.2014.05.024 25326742 PMC4637944

[25] Hong SJ Kim BK Shin DH Nam CM Kim JS Ko YG Effect of intravascular ultrasound-guided vs angiography-guided everolimus-eluting stent implantation: the IVUS-XPL randomized clinical trial. *JAMA.* (2015) 314:2155–63. 10.1001/jama.2015.15454 26556051

[26] Tan Q Wang Q Liu D Zhang S Zhang Y Li Y. Intravascular ultrasound-guided unprotected left main coronary artery stenting in the elderly. *Saudi Med J.* (2015) 36:549–53. 10.15537/smj.2015.5.11251 25935174 PMC4436750

[27] Wang HX Dong PS Li ZJ Wang HL Wang K Liu XY. Application of intravascular ultrasound in the emergency diagnosis and treatment of patients with ST-segment elevation myocardial infarction. *Echocardiography.* (2015) 32:1003–8. 10.1111/echo.12794 25287702

[28] Nguyen P Makris A Hennessy A Jayanti S Wang A Park K Standard versus ultrasound-guided radial and femoral access in coronary angiography and intervention (SURF): a randomised controlled trial. *EuroIntervention.* (2019) 15:e522–30. 10.4244/EIJ-D-19-00336 31113763

[29] Kala P Cervinka P Jakl M Kanovsky J Kupec A Spacek R OCT guidance during stent implantation in primary PCI: a randomized multicenter study with nine months of optical coherence tomography follow-up. *Int J Cardiol.* (2018) 250:98–103. 10.1016/j.ijcard.2017.10.059 29079414

[30] Ueki Y Yamaji K Barbato E Nef H Brugaletta S Alfonso F Randomized comparison of optical coherence tomography versus angiography to guide bioresorbable vascular scaffold implantation: the OPTICO BVS study. *Cardiovasc Revasc Med.* (2020) 21:1244–50. 10.1016/j.carrev.2020.03.023 32205067

[31] van Nunen LX Zimmermann FM Tonino PA Barbato E Baumbach A Engstrøm T Fractional flow reserve versus angiography for guidance of PCI in patients with multivessel coronary artery disease (FAME): 5-year follow-up of a randomised controlled trial. *Lancet.* (2015) 386:1853–60. 10.1016/S0140-6736(15)00057-4 26333474

[32] Zhang Z Li K Tian J. Efficacy and safety outcomes of fractional flow reserve in guiding clinical therapy of non-ST-segment elevation myocardial infarction compared with angiography alone in elderly Chinese patients. *Clin Interv Aging.* (2016) 11:1751–4. 10.2147/CIA.S123735 27932871 PMC5135069

[33] Puymirat E Cayla G Simon T Steg PG Montalescot G Durand-Zaleski I Multivessel PCI guided by FFR or angiography for myocardial infarction. *N Engl J Med.* (2021) 385:297–308. 10.1056/NEJMoa2104650 33999545

[34] Xu B Tu S Song L Jin Z Yu B Fu G Angiographic quantitative flow ratio-guided coronary intervention (FAVOR III China): a multicentre, randomised, sham-controlled trial. *Lancet.* (2021) 398:2149–59. 10.1016/S0140-6736(21)02248-0 34742368

[35] Habara M Nasu K Terashima M Kaneda H Yokota D Ko E Impact of frequency-domain optical coherence tomography guidance for optimal coronary stent implantation in comparison with intravascular ultrasound guidance. *Circ Cardiovasc Interv.* (2012) 5:193–201. 10.1161/circinterventions.111.965111 22456026

[36] Kubo T Shinke T Okamura T Hibi K Nakazawa G Morino Y Optical frequency domain imaging vs. intravascular ultrasound in percutaneous coronary intervention (OPINION trial): one-year angiographic and clinical results. *Eur Heart J.* (2017) 38:3139–47. 10.1093/eurheartj/ehx351 29121226 PMC5837511

[37] Muramatsu T Ozaki Y Nanasato M Ishikawa M Nagasaka R Ohota M Comparison between optical frequency domain imaging and intravascular ultrasound for percutaneous coronary intervention guidance in biolimus A9-eluting stent implantation: a randomized MISTIC-1 non-inferiority trial. *Circ Cardiovasc Interv.* (2020) 13:e009314. 10.1161/CIRCINTERVENTIONS.120.009314 33106049 PMC7665240

[38] Burzotta F Leone AM Aurigemma C Zambrano A Zimbardo G Arioti M Fractional flow reserve or optical coherence tomography to guide management of angiographically intermediate coronary stenosis: a single-center trial. *JACC Cardiovasc Interv.* (2020) 13:49–58. 10.1016/j.jcin.2019.09.034 31918942

[39] Ali ZA Karimi Galougahi K Maehara A Shlofmitz RA Fabbiocchi F Guagliumi G Outcomes of optical coherence tomography compared with intravascular ultrasound and with angiography to guide coronary stent implantation: one-year results from the ILUMIEN III: OPTIMIZE PCI trial. *EuroIntervention.* (2021) 16:1085–91. 10.4244/EIJ-D-20-00498 32540793 PMC9724851

[40] Witzenbichler B Maehara A Weisz G Neumann FJ Rinaldi MJ Metzger DC Relationship between intravascular ultrasound guidance and clinical outcomes after drug-eluting stents: the assessment of dual antiplatelet therapy with drug-eluting stents (ADAPT-DES) study. *Circulation.* (2014) 129:463–70. 10.1161/CIRCULATIONAHA.113.003942 24281330

[41] Räber L Mintz GS Koskinas KC Johnson TW Holm NR Onuma Y Clinical use of intracoronary imaging. Part 1: guidance and optimization of coronary interventions. An expert consensus document of the European association of percutaneous cardiovascular interventions. *Eur Heart J.* (2018) 39:3281–300. 10.1093/eurheartj/ehy285 29790954

[42] Ali ZA Karimi Galougahi K Nazif T Maehara A Hardy MA Cohen DJ Imaging- and physiology-guided percutaneous coronary intervention without contrast administration in advanced renal failure: a feasibility, safety, and outcome study. *Eur Heart J.* (2016) 37:3090–5. 10.1093/eurheartj/ehw078 26957421 PMC6279210

[43] Di Gioia G De Bruyne B Pellicano M Bartunek J Colaiori I Fiordelisi A Fractional flow reserve in patients with reduced ejection fraction. *Eur Heart J.* (2020) 41:1665–72. 10.1093/eurheartj/ehz571 31419282

[44] Ali Z Landmesser U Karimi Galougahi K Maehara A Matsumura M Shlofmitz RA Optical coherence tomography-guided coronary stent implantation compared to angiography: a multicentre randomised trial in PCI - design and rationale of ILUMIEN IV: OPTIMAL PCI. *EuroIntervention.* (2021) 16:1092–9. 10.4244/EIJ-D-20-00501 32863246 PMC9725042

[45] Kim JS Hong MK Shin DH Kim BK Ko YG Choi D Quantitative and qualitative changes in DES-related neointimal tissue based on serial OCT. *JACC Cardiovasc Imaging.* (2012) 5:1147–55. 10.1016/j.jcmg.2012.01.024 23153915

[46] Heeger CH Popescu SS Vogler J Eitel C Kuck KH Tilz RR. Single very high-power short-duration application for successful ablation of frequent premature ventricular contractions. *Europace.* (2022) 24:649. 10.1093/europace/euab288 34850885

[47] Santarpia G De Rosa S Polimeni A Giampà S Micieli M Curcio A Efficacy and safety of non-vitamin k antagonist oral anticoagulants versus vitamin K antagonist oral anticoagulants in patients undergoing radiofrequency catheter ablation of atrial fibrillation: a meta-analysis. *PLoS One.* (2015) 10:e0126512. 10.1371/journal.pone.0126512 25974377 PMC4431735

