# Supplementary material for: Clinical Outcomes Following Hemodynamic Parameter or Intravascular Imaging-Guided Percutaneous Coronary Intervention in the Era of Drug-Eluting Stents: An Updated Systematic Review and Bayesian Network Meta-Analysis of 28 Randomized Trials and 11,860 Patients
Source: Front Cardiovasc Med. 2022 Jun 3;9:860189. doi: 10.3389/fcvm.2022.860189 (PMC9203695; doi:10.3389/fcvm.2022.860189)
Supplement: Supplementary file 1 [file Data_Sheet_1.docx]

Supplement Table 1. Search strategy algorithm

| No. | Search terms | Records |
| --- | --- | --- |
| MEDLINE | | |
| 1 | coronary angiography or CAG or intravascular ultrasound or IVUS or optical coherence tomography or OCT or optical frequency domain imaging or OFDI or fractional flow reserve or FFR or instantaneous wave-free ratio or iFR or quantitative flow ratio or QFR | 113,291 |
| 2 | percutaneous coronary intervention or PCI | 46,837 |
| 3 | randomized controlled trial or randomized trial or trial | 2,685,523 |
| 4 | 1 and 2 and 3 | 389 |
| Cochrane Central Register of Controlled Trials | | |
| 5 | coronary angiography or CAG or intravascular ultrasound or IVUS or optical coherence tomography or OCT or optical frequency domain imaging or OFDI or fractional flow reserve or FFR or instantaneous wave-free ratio or iFR or quantitative flow ratio or QFR | 38,117 |
| 6 | percutaneous coronary intervention or PCI | 20,113 |
| 7 | 5 and 6 | 271 |
| EMBASE | | |
| 8 | coronary angiography or CAG or intravascular ultrasound or IVUS or optical coherence tomography or OCT or optical frequency domain imaging or OFDI or fractional flow reserve or FFR or instantaneous wave-free ratio or iFR or quantitative flow ratio or QFR | 146,281 |
| 9 | percutaneous coronary intervention or PCI | 98,450 |
| 10 | randomized controlled trial or randomized trial or trial | 2,023,151 |
| 11 | 8 and 9 and 10 | 301 |
| Web of Science | | |
| 12 | coronary angiography or CAG or intravascular ultrasound or IVUS or optical coherence tomography or OCT or optical frequency domain imaging or OFDI or fractional flow reserve or FFR or instantaneous wave-free ratio or iFR or quantitative flow ratio or QFR | 149,771 |
| 13 | percutaneous coronary intervention or PCI | 79,935 |
| 14 | randomized controlled trial or randomized trial or trial | 2,016,630 |
| 15 | 12 and 13 and 14 | 368 |

Supplementary Table 2. Definition of Clinical Outcome for Each Randomized Trial

| Study/First author, year | MACE | cardiovascular death | MI | TVR/TLR | stent thrombosis | any revascularization |
| --- | --- | --- | --- | --- | --- | --- |
| IVUS versus CAG |  |  |  |  |  |  |
| Jakabcin,^1^ 2010 | all-cause death, MI, TLR | cardiac origin | Q-wave MI: new, pathologic Q waves in two or more ECG leads, with post procedural CK levels of two times the URL and CK-MB >10% CK levels. Non-Q-wave MI: elevation of post procedural CK levels of two times the URL, with CK-MB above normal and no-Q-waves | TLR: reinterventions inside the stent implanted during the index procedure or within 5 mm proximal or distal to the stent | according to ARC | - |
| AVIO,^2^ 2013 | MI, cardiac death, TVR | - | Q-wave MI: pathological Q- waves post-procedure lasting at least 0.4s in two consecutive leads with an elevation of CK 2 times the URL. Non–Q-wave post-procedural MI: elevation of CK two times the URL with an elevated CK–MB 2 times the URL | TLR: revascularization occurred inside the implanted stent or within 5 mm proximally or distally, TVR: repeated intervention in the same vessel by PCI or CABG | according to ARC | - |
| RESET,^3^ 2013 | cardiac death, MI, stent thrombosis, TVR | all deaths were considered cardiovascular deaths unless a definite noncardiovascular cause was established | presence of clinical symptoms, ECG changes, or abnormal imaging findings of MI combined with an increase in CK-MB fraction to greater than 3 X the URL or an increase in troponin T/troponin I to more than the 99th percentile of the URL, unrelated to an interventional procedure | TVR: repeat PCI or CABG of the target vessel with either: 1) ischemic symptoms or a positive stress test and angiographic diameter stenosis ≥50% by quantitative coronary angiographic analysis; or 2) angiographic diameter stenosis 􏰁≥70% by quantitative coronary angiographic analysis without ischemic symptoms or a positive stress test | according to ARC | - |
| MOZART,^4^ 2014 | - | - | - | - | according to ARC | - |
| IVUS-XPL,^5^ 2015 | cardiac death, MI, or TLR | all deaths were considered cardiac deaths unless a definite noncardiac cause could be established | clinical symptoms, ECG changes, or abnormal imaging findings of MI, combined with an increase in the CK-MB fraction above the URL or an increase in troponin T or troponin I to a level greater than the 99th percentile of the URL | TLR: repeat PCI or CABG of the target lesions with either of the following: (1) symptoms of ischemia or a positive stress test and angiographic diameter stenosis of 50% or greater by quantitative coronary angiographic analysis, or (2) angiographic diameter stenosis of 70% or greater by quantitative coronary angiographic analysis without symptoms of ischemia or a positive stress test | according to ARC | - |
| CTO-IVUS,^6^ 2015 | cardiac death, MI, or TVR | all deaths were considered cardiac death unless an unequivocal noncardiac cause could be established | clinical symptoms, ECG changes, or abnormal imaging findings associated with MI combined with an increase in CK-MB above the URL or troponin T/I greater than the 99th percentile of the URL, unrelated to an interventional procedure | TVR: repeat PCI or CABG of the target vessel with ischemic symptoms or positive stress test results as well as lesion severity of >50% angiographic diameter stenosis by quantitative angiographic analysis | according to ARC | - |
| Tan,^7^ 2015 | death, MI, and TLR | deaths were classified as either cardiac or non-cardiac, deaths that could not be classified were considered cardiac according to the Academic Research Consortium definitions | an elevation of serum creatine kinase or troponin 3 times the URL, together with chest pain lasting more than 30 minutes | TVR: new intervention (PCI/CABG) on a previously implanted stent, or within 5 mm of that stent | according to ARC | - |
| AIR-CTO,^8^ 2015 | all-cause death, cardiac death, MI,TLR, and TVR | all deaths were determined to be cardiac in origin unless non-cardiac reasons were indicated | plasma level of troponin I/T increased to >3 times the URL in no fewer than two blood samples | TLR/TVR: any repeated revascularisation (PCI/CABG) for target lesions and target vessels, respectively, in the presence of symptoms or objective signs of ischaemia | according to ARC | - |
| Wang,^9^ 2015 | cardiac death, MI, TVR, and intractable myocardial ischemia | - | - | - | - | - |
| ULTIMATE,^10^ 2018 | all-cause death, MI, TLR, stroke | any death without a clear noncardiac cause | CK-MB >10 times the URL of the assay, or >5 times the URL plus either: 1) new pathological Q waves in >2 contiguous leads or new left bundle branch block; or 2) angiographically documented graft or coronary artery occlusion, or new severe stenosis with thrombosis; or 3) imaging evidence of new loss of viable myocardium or new regional wall motion abnormality | TVR: angina or ischemia referable to the target vessel requiring repeat PCI or CABG | according to ARC | - |
| SURF,^11^ 2019 | death, stroke, MI or TLR, and vascular complications | - | - | - | - | - |
| OCT/OFDI versus CAG | | | | | | |
| DOCTORS,^12^ 2016 | - | - | Third Universal Definition of Myocardial Infarction | - | - | - |
| ROBUST,^13^ 2018 | death, MI, and TLR | - | new, pathologic Q waves in two or more ECG leads, with post-procedural CK levels three times higher than the URL and CK-MB >10% of CK levels | TLR: revascularization within 5 mm to the stent edges (in-segment) on angiography | - | - |
| OPTICO BVS,^14^ 2020 | - | - | - | - | - | - |
| OPTIMUM,^15^ 2020 | - | - | - | - | - | - |
| FFR/QFR versus CAG | | | | | | |
| FAME,^16^ 2015 | death, MI, and any revascularisation | death from an unknown cause was also designated as cardiac death | threefold or greater elevation of CK-MB level or new Q-waves in ≥2 contiguous leads of the ECG | - | - | - |
| DKCRUSH-VI,^17^ 2015 | cardiac death, MI, or TVR | all deaths were considered cardiac in origin unless noncardiac causes were certain | CK-MB increase to more than 3 times the URL in ≥2 samples | - | according to ARC | - |
| FAMOUS–NSTEMI,^18^ 2015 | cardiac death or hospitalization for MI or heart failure | - | - | - | - | PCI and CABG |
| DEFER-DES,^19^ 2015 | cardiac death, MI, and TLR | all deaths were considered cardiac unless there was a clear noncardiac cause | levated cardiac enzyme with ischemic symptoms or new pathological Q waves on ECG | - | - | - |
| Zhang,^20^ 2016 | cardiovascular death, MI, or unplanned hospitalization for heart failure | - | - | - | - | - |
| Quintella,^21^ 2019 | - | - | -- | - | - | - |
| FLOWER-MI,^22^ 2021 | death, MI, or unplanned hospitalization leading to urgent revascularization | all deaths were considered cardiac unless an unequivocal non-cardiac cause can be established | evidence of myocardial  necrosis in a clinical setting consistent with myocardial ischemia | TLR: ischemia-driven repeat PCI of the target lesion or CABG of the  target vessel. TVR: ischemia-driven repeat PCI or CABG in any lesion of the target vessel | according to ARC | PCI or CABG |
| FAVOR III China,^23^ 2021 | cardiac death, MI, and TVR | all deaths were considered cardiac in origin unless noncardiac causes were certain | CK-MB increase to more than 3 times the URL in ≥2 samples | - | according to ARC | - |
| OCT/OFDI versus IVUS | | | | | | |
| Habara,^24^ 2012 | - | - | CK elevation to 2 times normal | - | - | - |
| OPINION,^25^ 2017 | cardiac death, MI, or TLR | any death due to proximate cardiac cause, unwitnessed death and death of unknown cause, all procedure related deaths including those related to concomitant treatment | ST elevation or a new abnormal Q wave on ECG; myocardial infarction-specific clinical symptom; and the serum level of troponin or CK-MB higher than the URL | TVR: unplanned repeat PCI or CABG for a stenosis in another part of the vessel treated at the index PCI. TLR: repeat PCI or CABG for restenosis or other complications at the lesion treated during index PCI, or occurring within 5 mm of the PCI site | according to ARC | - |
| MISTIC-1,^26^ 2020 | cardiovascular mortality, MI, or TLR | any death due to clinically relevant cardiac or  noncoronary vascular causes, or unexpected intrinsic death unless an unequivocal  noncardiac cause could be established | third universal definition of MI | - | according to ARC | - |
| OCT versus FFR |  |  |  |  |  |  |
| FORZA,^27^ 2020 | death, MI, and TVR | - | third universal definition of MI | TVR: clinically driven revascularization by either PCI or CABG on the vessel evaluated using fractional flow reserve or optical coherence tomography, regardless of the fact that it was initially treated by PCI | - | - |
| OCT versus IVUS versus CAG | | | | | | |
| ILUMIEN III,^28^ 2021 | death, MI, stent thrombosis, or repeat revascularisation | - | - | - | - | - |

ARC: Academic Research Consortium; CABG: coronary artery bypass graft; CAG: coronary angiography; CK: creatine kinase; ECG: electrocardiograph; FFR: fractional flow reserve; IVUS: intravascular ultrasound; MACE: major adverse cardiovascular events; MI: myocardial infarction; OCT: optical coherence tomography; OFDI: optical frequency domain imaging; PCI: percutaneous coronary intervention; QFR: quantitative flow ratio; TVR/TLR: target vessel/lesion revascularization; URL: upper reference limit

References

1. Jakabcin J, Spacek R, Bystron M, Kvasnák M, Jager J, Veselka J, Kala P and Cervinka P. Long-term health outcome and mortality evaluation after invasive coronary treatment using drug eluting stents with or without the IVUS guidance. Randomized control trial. HOME DES IVUS. *Catheter Cardiovasc Interv*. 2010;75:578-83.

2. Chieffo A, Latib A, Caussin C, Presbitero P, Galli S, Menozzi A, Varbella F, Mauri F, Valgimigli M, Arampatzis C, Sabate M, Erglis A, Reimers B, Airoldi F, Laine M, Palop RL, Mikhail G, Maccarthy P, Romeo F and Colombo A. A prospective, randomized trial of intravascular-ultrasound guided compared to angiography guided stent implantation in complex coronary lesions: the AVIO trial. *Am Heart J*. 2013;165:65-72.

3. Kim JS, Kang TS, Mintz GS, Park BE, Shin DH, Kim BK, Ko YG, Choi D, Jang Y and Hong MK. Randomized comparison of clinical outcomes between intravascular ultrasound and angiography-guided drug-eluting stent implantation for long coronary artery stenoses. *JACC Cardiovasc Interv*. 2013;6:369-76.

4. Mariani J, Jr., Guedes C, Soares P, Zalc S, Campos CM, Lopes AC, Spadaro AG, Perin MA, Filho AE, Takimura CK, Ribeiro E, Kalil-Filho R, Edelman ER, Serruys PW and Lemos PA. Intravascular ultrasound guidance to minimize the use of iodine contrast in percutaneous coronary intervention: the MOZART (Minimizing cOntrast utiliZation With IVUS Guidance in coRonary angioplasTy) randomized controlled trial. *JACC Cardiovasc Interv*. 2014;7:1287-93.

5. Hong SJ, Kim BK, Shin DH, Nam CM, Kim JS, Ko YG, Choi D, Kang TS, Kang WC, Her AY, Kim YH, Hur SH, Hong BK, Kwon H, Jang Y and Hong MK. Effect of Intravascular Ultrasound-Guided vs Angiography-Guided Everolimus-Eluting Stent Implantation: The IVUS-XPL Randomized Clinical Trial. *Jama*. 2015;314:2155-63.

6. Kim BK, Shin DH, Hong MK, Park HS, Rha SW, Mintz GS, Kim JS, Kim JS, Lee SJ, Kim HY, Hong BK, Kang WC, Choi JH and Jang Y. Clinical Impact of Intravascular Ultrasound-Guided Chronic Total Occlusion Intervention With Zotarolimus-Eluting Versus Biolimus-Eluting Stent Implantation: Randomized Study. *Circ Cardiovasc Interv*. 2015;8:e002592.

7. Tan Q, Wang Q, Liu D, Zhang S, Zhang Y and Li Y. Intravascular ultrasound-guided unprotected left main coronary artery stenting in the elderly. *Saudi Med J*. 2015;36:549-53.

8. Tian NL, Gami SK, Ye F, Zhang JJ, Liu ZZ, Lin S, Ge Z, Shan SJ, You W, Chen L, Zhang YJ, Mintz G and Chen SL. Angiographic and clinical comparisons of intravascular ultrasound- versus angiography-guided drug-eluting stent implantation for patients with chronic total occlusion lesions: two-year results from a randomised AIR-CTO study. *EuroIntervention*. 2015;10:1409-17.

9. Wang HX, Dong PS, Li ZJ, Wang HL, Wang K and Liu XY. Application of Intravascular Ultrasound in the Emergency Diagnosis and Treatment of Patients with ST-Segment Elevation Myocardial Infarction. *Echocardiography*. 2015;32:1003-8.

10. Zhang J, Gao X, Kan J, Ge Z, Han L, Lu S, Tian N, Lin S, Lu Q, Wu X, Li Q, Liu Z, Chen Y, Qian X, Wang J, Chai D, Chen C, Li X, Gogas BD, Pan T, Shan S, Ye F and Chen SL. Intravascular Ultrasound Versus Angiography-Guided Drug-Eluting Stent Implantation: The ULTIMATE Trial. *J Am Coll Cardiol*. 2018;72:3126-3137.

11. Nguyen P, Makris A, Hennessy A, Jayanti S, Wang A, Park K, Chen V, Nguyen T, Lo S, Xuan W, Leung M and Juergens C. Standard versus ultrasound-guided radial and femoral access in coronary angiography and intervention (SURF): a randomised controlled trial. *EuroIntervention*. 2019;15:e522-e530.

12. Meneveau N, Souteyrand G, Motreff P, Caussin C, Amabile N, Ohlmann P, Morel O, Lefrançois Y, Descotes-Genon V, Silvain J, Braik N, Chopard R, Chatot M, Ecarnot F, Tauzin H, Van Belle E, Belle L and Schiele F. Optical Coherence Tomography to Optimize Results of Percutaneous Coronary Intervention in Patients with Non-ST-Elevation Acute Coronary Syndrome: Results of the Multicenter, Randomized DOCTORS Study (Does Optical Coherence Tomography Optimize Results of Stenting). *Circulation*. 2016;134:906-17.

13. Kala P, Cervinka P, Jakl M, Kanovsky J, Kupec A, Spacek R, Kvasnak M, Poloczek M, Cervinkova M, Bezerra H, Valenta Z, Attizzani GF, Schnell A, Hong L and Costa MA. OCT guidance during stent implantation in primary PCI: A randomized multicenter study with nine months of optical coherence tomography follow-up. *Int J Cardiol*. 2018;250:98-103.

14. Ueki Y, Yamaji K, Barbato E, Nef H, Brugaletta S, Alfonso F, Hill J, Cook S, Burzotta F, Karagiannis A, Windecker S and Räber L. Randomized Comparison of Optical Coherence Tomography Versus Angiography to Guide Bioresorbable Vascular Scaffold Implantation: The OPTICO BVS Study. *Cardiovasc Revasc Med*. 2020;21:1244-1250.

15. Onuma Y, Kogame N, Sotomi Y, Miyazaki Y, Asano T, Takahashi K, Kawashima H, Ono M, Katagiri Y, Kyono H, Nakatani S, Muramatsu T, Sharif F, Ozaki Y, Serruys PW and Okamura T. A Randomized Trial Evaluating Online 3-Dimensional Optical Frequency Domain Imaging-Guided Percutaneous Coronary Intervention in Bifurcation Lesions. *Circ Cardiovasc Interv*. 2020;13:e009183.

16. van Nunen LX, Zimmermann FM, Tonino PA, Barbato E, Baumbach A, Engstrøm T, Klauss V, MacCarthy PA, Manoharan G, Oldroyd KG, Ver Lee PN, Van't Veer M, Fearon WF, De Bruyne B and Pijls NH. Fractional flow reserve versus angiography for guidance of PCI in patients with multivessel coronary artery disease (FAME): 5-year follow-up of a randomised controlled trial. *Lancet*. 2015;386:1853-60.

17. Chen SL, Ye F, Zhang JJ, Xu T, Tian NL, Liu ZZ, Lin S, Shan SJ, Ge Z, You W, Liu YQ, Qian XS, Li F, Yang S, Kwan TW, Xu B and Stone GW. Randomized Comparison of FFR-Guided and Angiography-Guided Provisional Stenting of True Coronary Bifurcation Lesions: The DKCRUSH-VI Trial (Double Kissing Crush Versus Provisional Stenting Technique for Treatment of Coronary Bifurcation Lesions VI). *JACC Cardiovasc Interv*. 2015;8:536-46.

18. Layland J, Oldroyd KG, Curzen N, Sood A, Balachandran K, Das R, Junejo S, Ahmed N, Lee MM, Shaukat A, O'Donnell A, Nam J, Briggs A, Henderson R, McConnachie A and Berry C. Fractional flow reserve vs. angiography in guiding management to optimize outcomes in non-ST-segment elevation myocardial infarction: the British Heart Foundation FAMOUS-NSTEMI randomized trial. *Eur Heart J*. 2015;36:100-11.

19. Park SH, Jeon KH, Lee JM, Nam CW, Doh JH, Lee BK, Rha SW, Yoo KD, Jung KT, Cho YS, Lee HY, Youn TJ, Chung WY and Koo BK. Long-Term Clinical Outcomes of Fractional Flow Reserve-Guided Versus Routine Drug-Eluting Stent Implantation in Patients With Intermediate Coronary Stenosis: Five-Year Clinical Outcomes of DEFER-DES Trial. *Circ Cardiovasc Interv*. 2015;8:e002442.

20. Zhang Z, Li K and Tian J. Efficacy and safety outcomes of fractional flow reserve in guiding clinical therapy of non-ST-segment elevation myocardial infarction compared with angiography alone in elderly Chinese patients. *Clin Interv Aging*. 2016;11:1751-1754.

21. Quintella EF, Ferreira E, Azevedo VMP, Araujo DV, Sant'Anna FM, Amorim B and Albuquerque DC. Clinical Outcomes and Cost-Effectiveness Analysis of FFR Compared with Angiography in Multivessel Disease Patient. *Arq Bras Cardiol*. 2019;112:40-47.

22. Puymirat E, Cayla G, Simon T, Steg PG, Montalescot G, Durand-Zaleski I, le Bras A, Gallet R, Khalife K, Morelle JF, Motreff P, Lemesle G, Dillinger JG, Lhermusier T, Silvain J, Roule V, Labèque JN, Rangé G, Ducrocq G, Cottin Y, Blanchard D, Charles Nelson A, De Bruyne B, Chatellier G and Danchin N. Multivessel PCI Guided by FFR or Angiography for Myocardial Infarction. *N Engl J Med*. 2021;385:297-308.

23. Xu B, Tu S, Song L, Jin Z, Yu B, Fu G, Zhou Y, Wang J, Chen Y, Pu J, Chen L, Qu X, Yang J, Liu X, Guo L, Shen C, Zhang Y, Zhang Q, Pan H, Fu X, Liu J, Zhao Y, Escaned J, Wang Y, Fearon WF, Dou K, Kirtane AJ, Wu Y, Serruys PW, Yang W, Wijns W, Guan C, Leon MB, Qiao S and Stone GW. Angiographic quantitative flow ratio-guided coronary intervention (FAVOR III China): a multicentre, randomised, sham-controlled trial. *Lancet*. 2021.

24. Habara M, Nasu K, Terashima M, Kaneda H, Yokota D, Ko E, Ito T, Kurita T, Tanaka N, Kimura M, Ito T, Kinoshita Y, Tsuchikane E, Asakura K, Asakura Y, Katoh O and Suzuki T. Impact of frequency-domain optical coherence tomography guidance for optimal coronary stent implantation in comparison with intravascular ultrasound guidance. *Circ Cardiovasc Interv*. 2012;5:193-201.

25. Kubo T, Shinke T, Okamura T, Hibi K, Nakazawa G, Morino Y, Shite J, Fusazaki T, Otake H, Kozuma K, Ioji T, Kaneda H, Serikawa T, Kataoka T, Okada H and Akasaka T. Optical frequency domain imaging vs. intravascular ultrasound in percutaneous coronary intervention (OPINION trial): one-year angiographic and clinical results. *Eur Heart J*. 2017;38:3139-3147.

26. Muramatsu T, Ozaki Y, Nanasato M, Ishikawa M, Nagasaka R, Ohota M, Hashimoto Y, Yoshiki Y, Takatsu H, Ito K, Kamiya H, Yoshida Y, Murohara T and Izawa H. Comparison Between Optical Frequency Domain Imaging and Intravascular Ultrasound for Percutaneous Coronary Intervention Guidance in Biolimus A9-Eluting Stent Implantation: A Randomized MISTIC-1 Non-Inferiority Trial. *Circ Cardiovasc Interv*. 2020;13:e009314.

27. Burzotta F, Leone AM, Aurigemma C, Zambrano A, Zimbardo G, Arioti M, Vergallo R, De Maria GL, Cerracchio E, Romagnoli E, Trani C and Crea F. Fractional Flow Reserve or Optical Coherence Tomography to Guide Management of Angiographically Intermediate Coronary Stenosis: A Single-Center Trial. *JACC Cardiovasc Interv*. 2020;13:49-58.

28. Ali ZA, Karimi Galougahi K, Maehara A, Shlofmitz RA, Fabbiocchi F, Guagliumi G, Alfonso F, Akasaka T, Matsumura M, Mintz GS, Ben-Yehuda O, Zhang Z, Rapoza RR, West NEJ and Stone GW. Outcomes of optical coherence tomography compared with intravascular ultrasound and with angiography to guide coronary stent implantation: one-year results from the ILUMIEN III: OPTIMIZE PCI trial. *EuroIntervention*. 2021;16:1085-1091.
