# Supplementary figures and images for: Clinical Outcomes Following Hemodynamic Parameter or Intravascular Imaging-Guided Percutaneous Coronary Intervention in the Era of Drug-Eluting Stents: An Updated Systematic Review and Bayesian Network Meta-Analysis of 28 Randomized Trials and 11,860 Patients
Source: Front Cardiovasc Med. 2022 Jun 3;9:860189. doi: 10.3389/fcvm.2022.860189 (PMC9203695; doi:10.3389/fcvm.2022.860189)

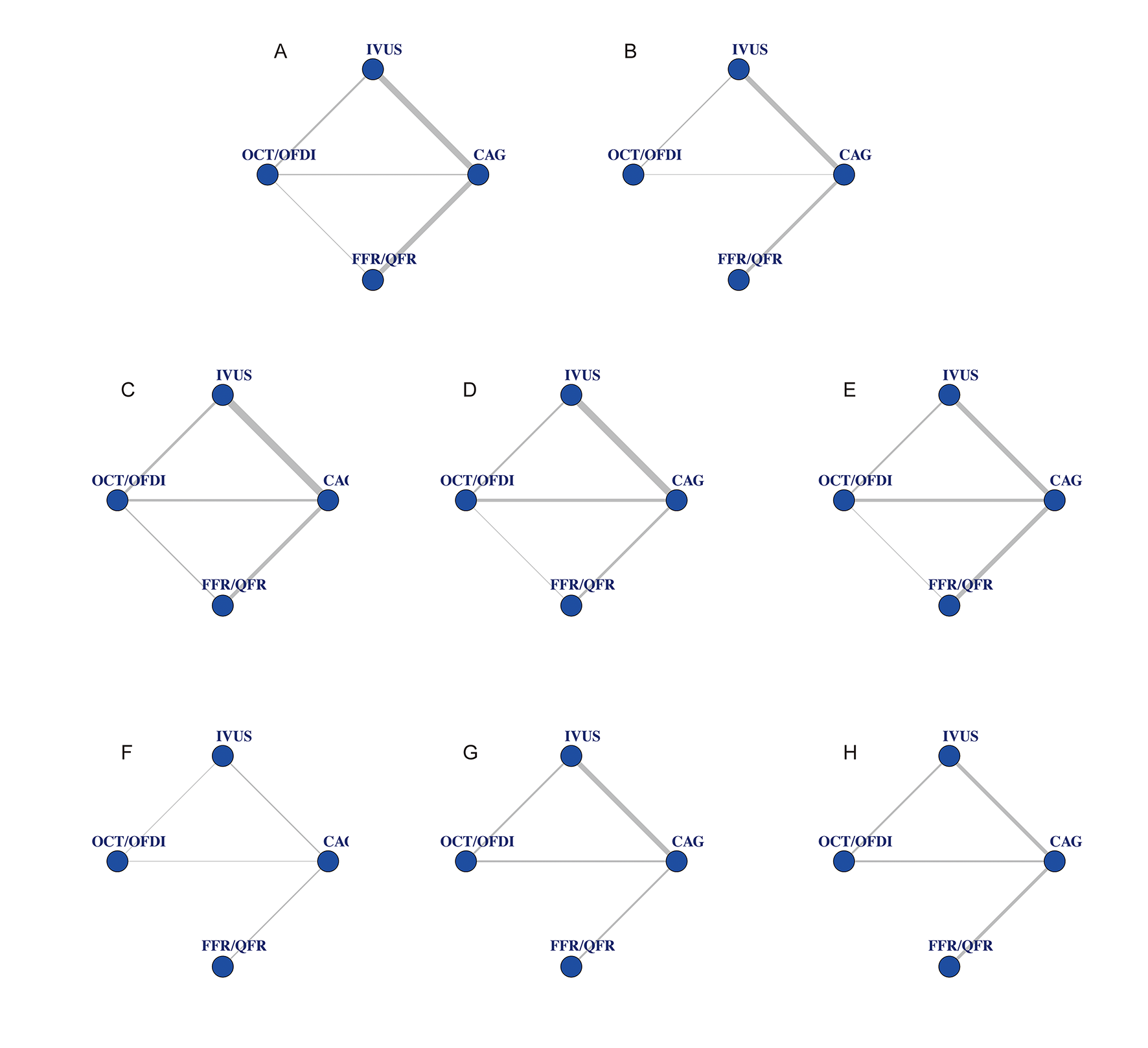

Supplement: Supplementary Figure 1 — Network evidence plot for major adverse cardiovascular events (A), Cardiovascular death (B), Myocardial infarction (C), Target vessel/lesion revascularization (D), All-cause death (E), Stroke (F), Stent thrombosis (G), and Any revascularization (H). CAG, coronary angiography; FFR, fractional flow reserve; IVUS, intravascular ultrasound; OCT, optical coherence tomography; OFDI, optical frequency domain imaging; QFR, quantitative flow ratio. [file Image_1.TIF]

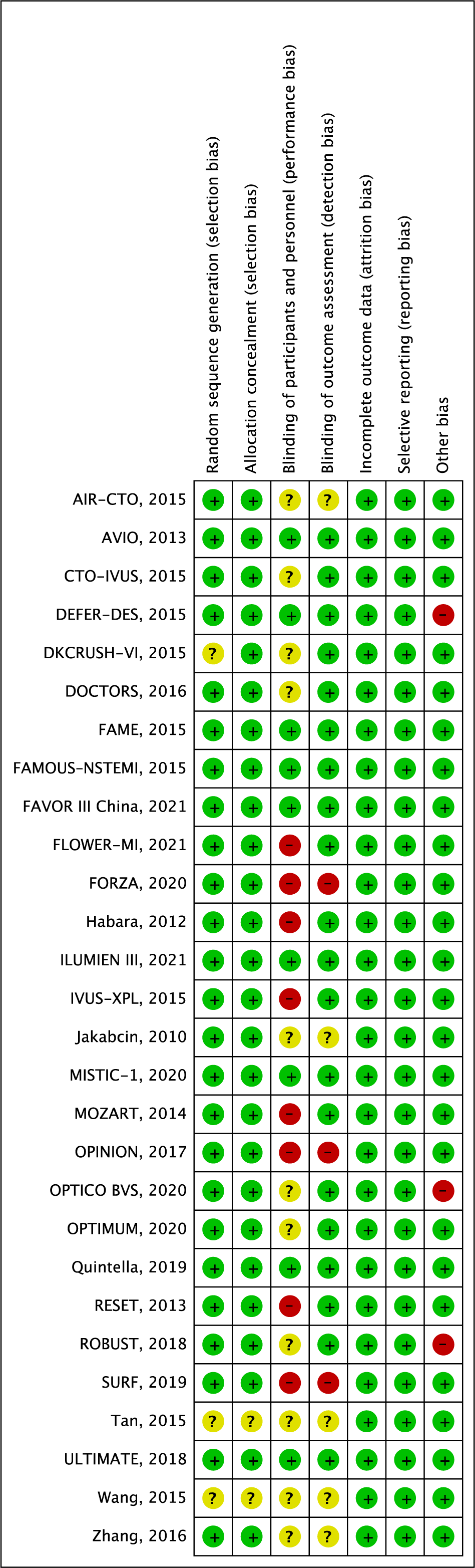

Supplement: Supplementary Figure 2 — Methodological quality assessment for each included randomized trial. +, low risk of bias; -, high risk of bias; ?, unclear risk of bias. [file Image_2.TIF]

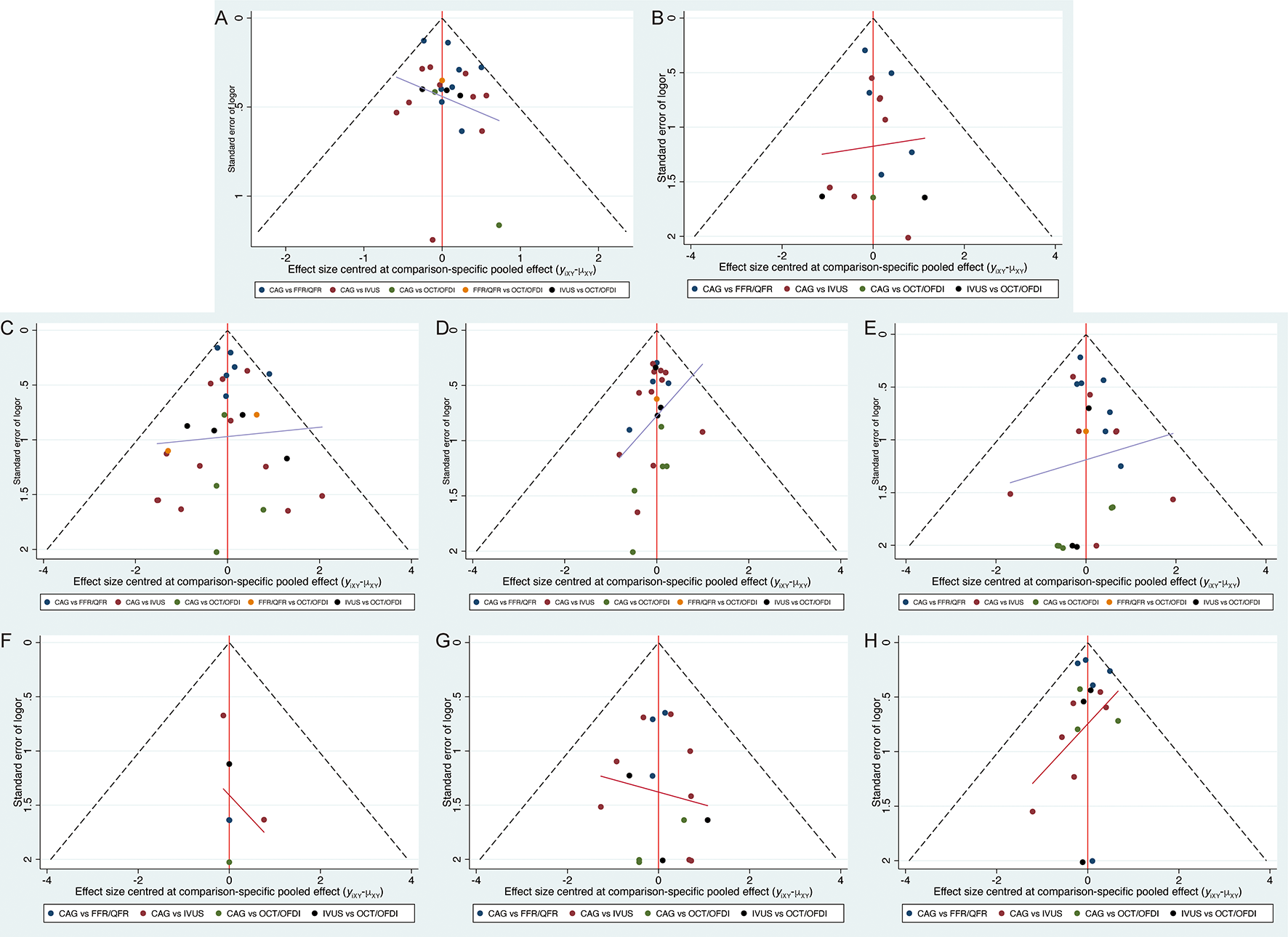

Supplement: Supplementary Figure 3 — Funnel plot of publication bias for major adverse cardiovascular events (A), Cardiovascular death (B), Myocardial infarction (C), Target vessel/lesion revascularization (D), All-cause death (E), Stroke (F), Stent thrombosis (G), and Any revascularization (H). CAG, coronary angiography; FFR, fractional flow reserve; IVUS, intravascular ultrasound; OCT, optical coherence tomography; OFDI, optical frequency domain imaging; QFR, quantitative flow ratio. [file Image_3.TIF]

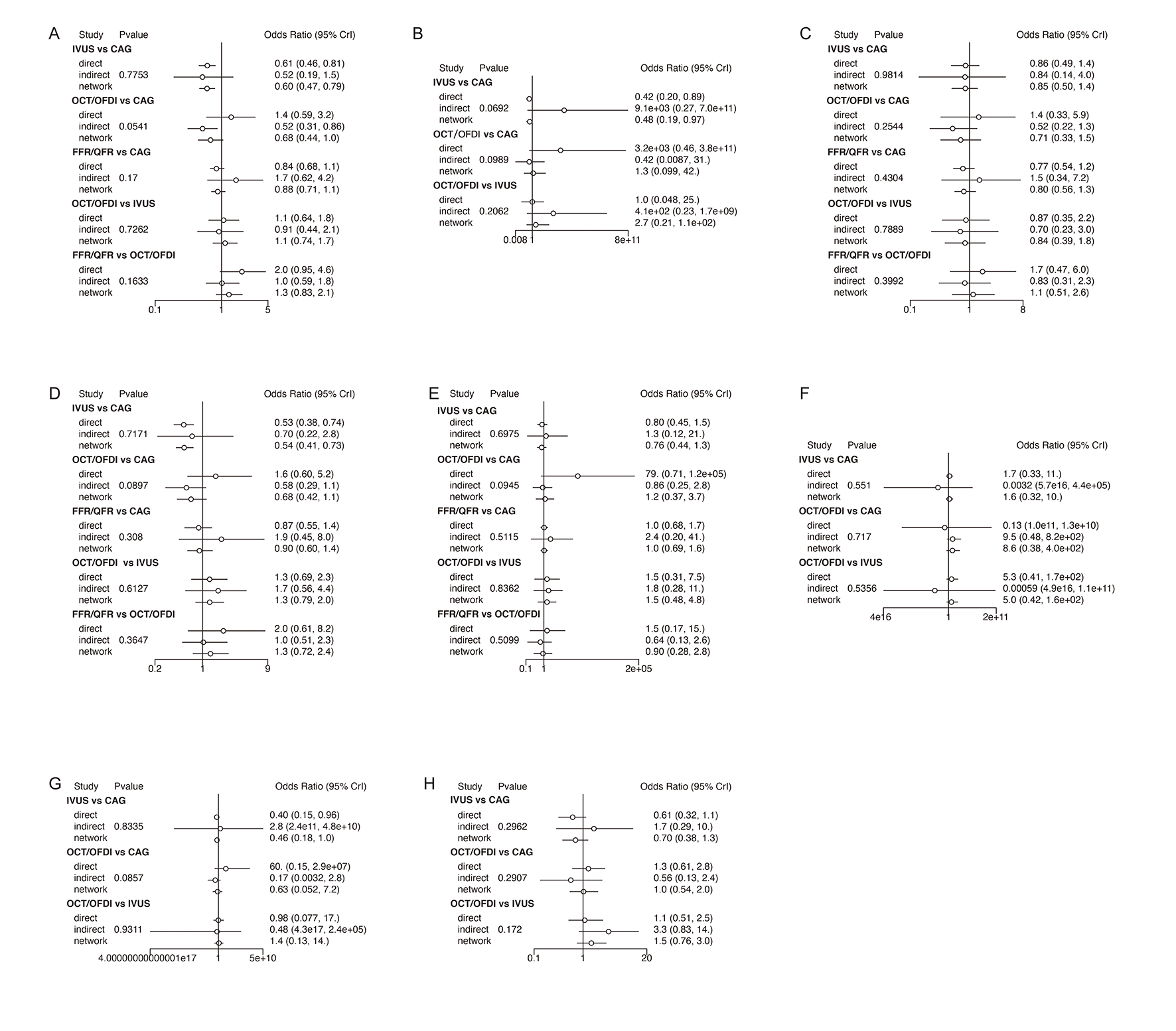

Supplement: Supplementary Figure 4 — Network node-split for major adverse cardiovascular events (A), Cardiovascular death (B), Myocardial infarction (C), Target vessel/lesion revascularization (D), All-cause death (E), Stroke (F), Stent thrombosis (G), and Any revascularization (H). CAG, coronary angiography; FFR, fractional flow reserve; IVUS, intravascular ultrasound; OCT, optical coherence tomography; OFDI, optical frequency domain imaging; QFR, quantitative flow ratio. [file Image_4.TIF]

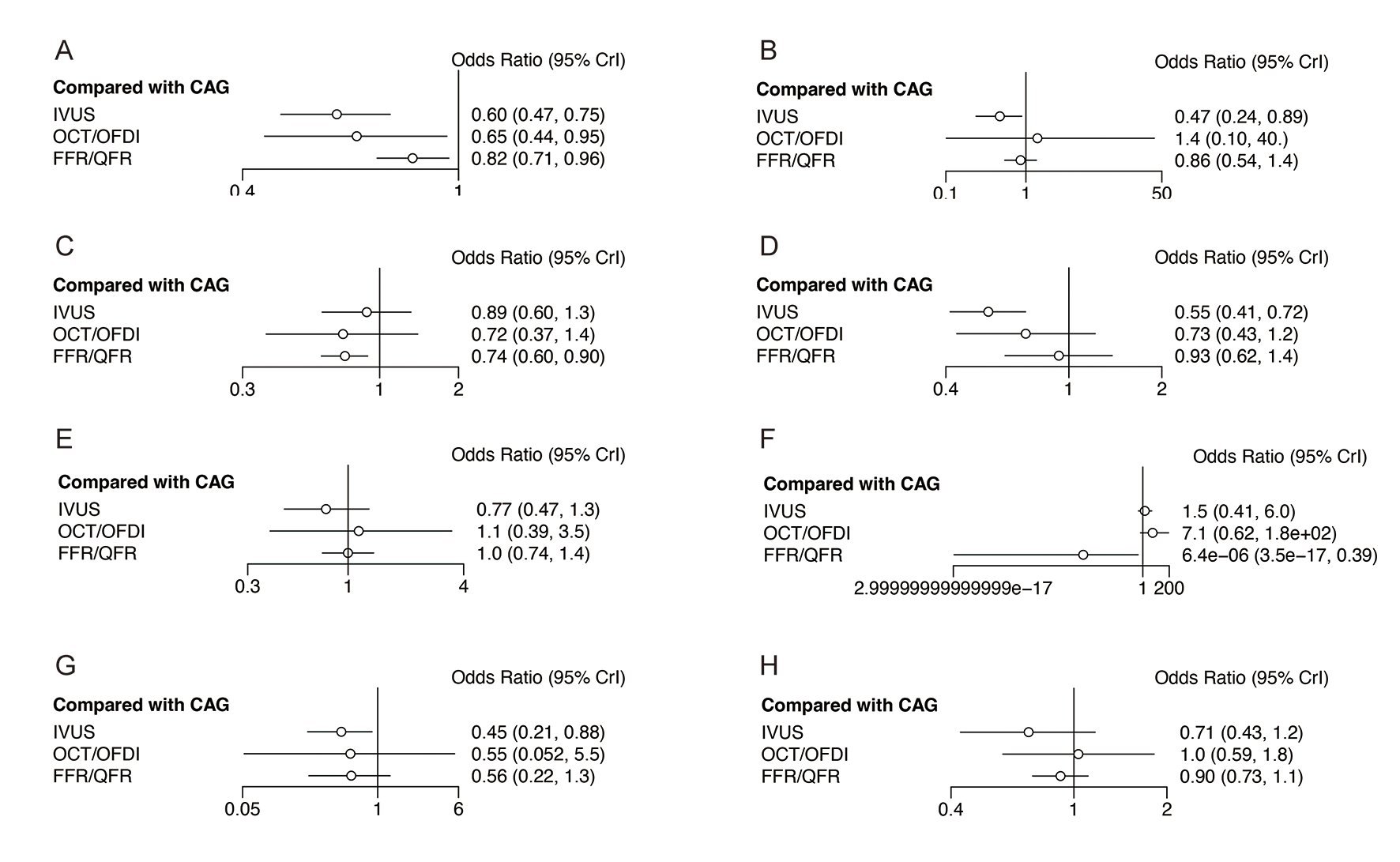

Supplement: Supplementary Figure 5 — Forest plot for major adverse cardiovascular events (A), Cardiovascular death (B), Myocardial infarction (C), Target vessel/lesion revascularization (D), All-cause death (E), Stroke (F), Stent thrombosis (G), and Any revascularization (H) with a fixed effects mode. CAG, coronary angiography; FFR, fractional flow reserve; IVUS, intravascular ultrasound; OCT, optical coherence tomography; OFDI, optical frequency domain imaging; QFR, quantitative flow ratio. [file Image_5.TIF]
